# The complete chloroplast genome of *Viola vaginata* (Violaceae), an endemic species of the snowy region in Japan

**DOI:** 10.1080/23802359.2024.2444595

**Published:** 2024-12-24

**Authors:** Yayoi Takahashi, Masato Fujiwara, Masaaki Ozeki, Masayuki U. Saito, Takaya Iwasaki

**Affiliations:** a Graduate School of Humanities and Sciences, Ochanomizu University, Tokyo, Japan; b Hyogo Prefectural Ono High School, Ono City, Hyogo, Japan; c Nagano Environmental Conservation Research Institute, Nagano City, Nagano, Japan; d Faculty of Agriculture, Yamagata University, Tsuruoka City, Yamagata, Japan

**Keywords:** Chloroplast genome, Violaceae, *Viola vaginata*, endemic, Japan

## Abstract

*Viola vaginata*, a perennial herb in subsection *Stolonosae*, is endemic to the snowy mountainous regions on the Sea of Japan side of Japan. Its complete chloroplast genome was 156,056 bp in length, comprising one large single-copy region (86,407 bp), one small single-copy region (17,301 bp), and two inverted repeat regions (27,174 bp each). It contained 111 unique genes, including 77 protein-coding genes, 30 transfer RNA genes, and 4 ribosomal RNA genes. Phylogenetic analysis placed *V. vaginata* in a clade with subsection *Biobatae* species and some *Patellares* species, while other *Patellares* species formed a distinct clade, contrasting with previous nuclear ITS results. These findings highlight the phylogenetic complexity within *Viola.*

## Introduction

**Figure 1.**
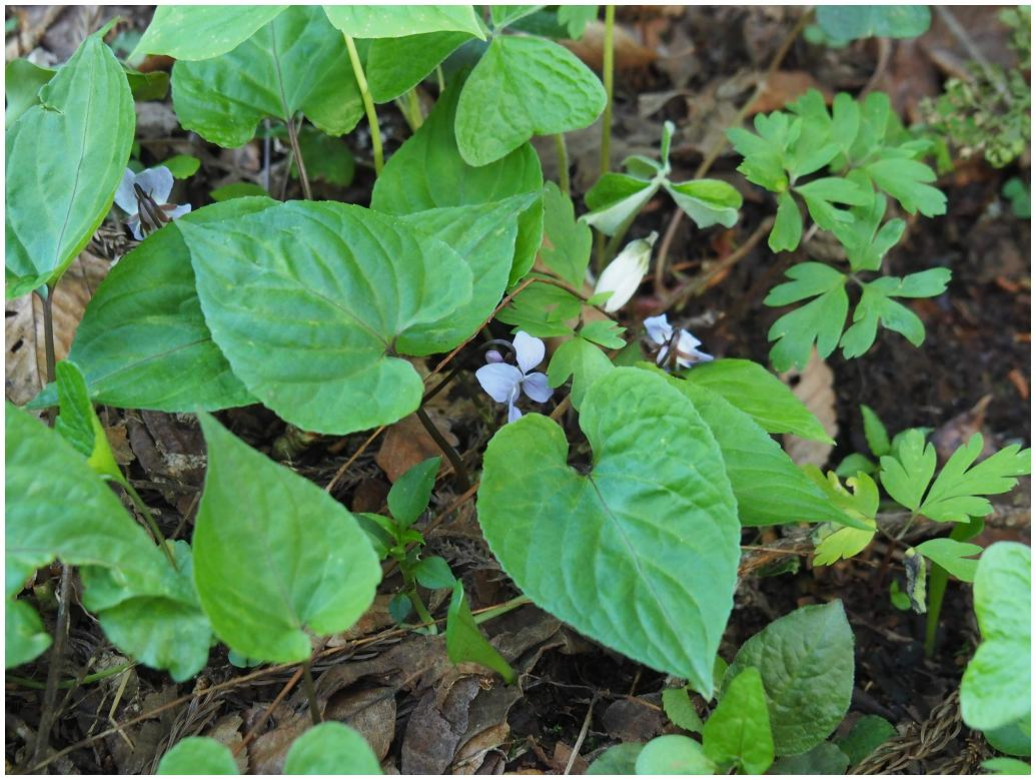
Image of *Viola vaginata* in bloom. This species is a perennial featuring light purple flowers, with hairless side petals and a thick, short spur. It has shiny, heart-shaped leaves with tip-pointed edges and sunken veins. The rhizomes are thick and long. This photograph was taken by the first author, Takaya Iwasaki, on 11 May 2019, in Tsuchidaru, Yuzawa, Niigata Prefecture, Japan. It is provided here free of any copyright restrictions.

The genus *Viola* L. 1753 (Violaceae) is one of the largest angiosperm genera, with approximately 525 to 664 species predominantly found in temperate regions worldwide (Clausen 1964; Ballard et al. 1998; Marcussen et al. 2022). In Japan, about 55 *Viola* species are known, including 14 endemic species (Kato and Ebihara 2011). The perennial herb *Viola vaginata* Maxim. 1877 (Figure 1), endemic to the snowy mountainous regions on the Sea of Japan side of Japan, was classified in section *Vaginatae* by Hama (2002) based on its chromosome count (*n* = 12), stemless form, and distinctive style shape. Globally, taxonomic classifications of section *Vaginatae* based on morphology and chromosome numbers have varied, often placed as subgenus *Vaginatae* within section *Nomimium*, subgenus *Adnatae* within section *Plagiostigma*, or subsection *Stolonosae* within section *Plagiostigma* (reviewed in Ballard et al. 1998). Recent detailed classifications leveraging morphology, chromosome counts, polyploidy, and nuclear ITS phylogenies have resolved much of the past ambiguity, placing this group within subsection *Stolonosae* within section *Plagiostigma* (Marcussen et al. 2022). Additionally, phylogenetic studies using complete chloroplast genomes, which provide essential phylogenetic insights and are maternally inherited, have been conducted for several species within section *Plagiostigma* (Kwak 2021; Cao et al. 2022; Moon and Kim 2023). Yet, no chloroplast genome from subsection *Stolonosae* has been sequenced, leaving its maternal phylogenetic position unclear. The study aims to construct the complete chloroplast genome of *V. vaginata* from subsection *Stolonosae* and clarify its phylogenetic relationships within *Viola*.

## Material and method

Plant material of *V. vaginata* was collected at Nishiaraya, Tsuruoka City, Yamagata Prefecture, Japan (latitude 38.6468° N, and longitude 139.8214° E) following local regulations and with permission. The specimen was deposited at the Makino Herbarium of Tokyo Metropolitan University (https://www.biol.se.tmu.ac.jp/herbarium/) (contact person: Noriaki Murakami, nmurak@tmu.ac.jp) under voucher number MAK-470660. Genomic DNA was extracted from the dried leaf tissue of *V. vaginata* using the Wizard Genomic DNA Purification Kit from Promega (Madison, WI, USA). Before the standard DNA extraction procedure of the kit, we performed two washes with PVP-HEPES buffer (pH 8.0 HEPES buffer, polyvinylpyrrolidone [PVP], L-ascorbic acid, 2-mercaptoethanol) (Setoguchi and Ohba 1995). PVP adsorbs polyphenols, thus inhibiting DNA-polyphenol binding (Echevarría-Machado et al. 2005). Whole-genome sequencing was conducted on the DNBSEQ platform (BGI, Hong Kong) with paired-end reads of 150 bp.

Sequence data were processed for adapter trimming and quality filtering with fastp v.0.20.0 (Chen et al. 2018) with default settings. De novo assembly of the chloroplast genome was achieved with NOVOPlasty v.4.3.1 (Dierckxsens et al. 2017) (Type; chloro, Genome range; 120,000–180,000, K-mer; 39), using *Viola verecunda* A.Gray 1858 (GenBank accession number MW586692) as the seed sequence. Sequence integrity was verified by mapping trimmed reads back to the assembled genome and assessing coverage depth with Python scripts (Ni et al. 2023). Discrepancies in initial annotations performed with GeSeq (Tillich et al. 2017) and CPGAVAS2 (Shi et al. 2019) were manually corrected, informed by annotation results from closely related *Viola* species. The assembly produced two versions of the chloroplast genome; the difference was the orientation of the SSC region, with one version presenting an inverted SSC sequence. Both sequences were annotated, and we selected the sequence homologous to that of *V. verecunda*. The software CPGView (Liu et al. 2023, http://www.1kmpg.cn/cpgview/) was utilized to draw the structural characteristics of the chloroplast genome and visualize the intron-containing genes.

To determine the phylogenetic position of *V. vaginata*, we obtained complete chloroplast genome sequences for 21 *Viola* species from GenBank. We also retrieved the chloroplast genome sequence of *Balanops balansae* Baill. 1873 (Balanopaceae) from GenBank as the outgroup. The chloroplast genome sequences were aligned using MAFFT v. 7.490 (Katoh and Standley 2013) with default parameters in Geneious Prime 2022.1.1 (https://www.geneious.com). Because the alignment results from MAFFT contained many gaps, the poorly aligned regions and gaps were removed using Gblocks v.0.91.1 (Castresana 2000; available at https://ngphylogeny.fr/tools/tool/276/form) with default parameters. The best-fit model for nucleotide substitution was determined based on AIC using ModelTest-NG v.0.1.7 (Darriba et al. 2020). A maximum likelihood (ML) phylogenetic tree was then constructed with RAxML-NG v.1.1.0 (Kozlov et al. 2019), employing the TVM+I + G4 model for the nucleotide substitution and evaluating branch support with 1,000 bootstrap replicates.

## Results

**Figure 2.**
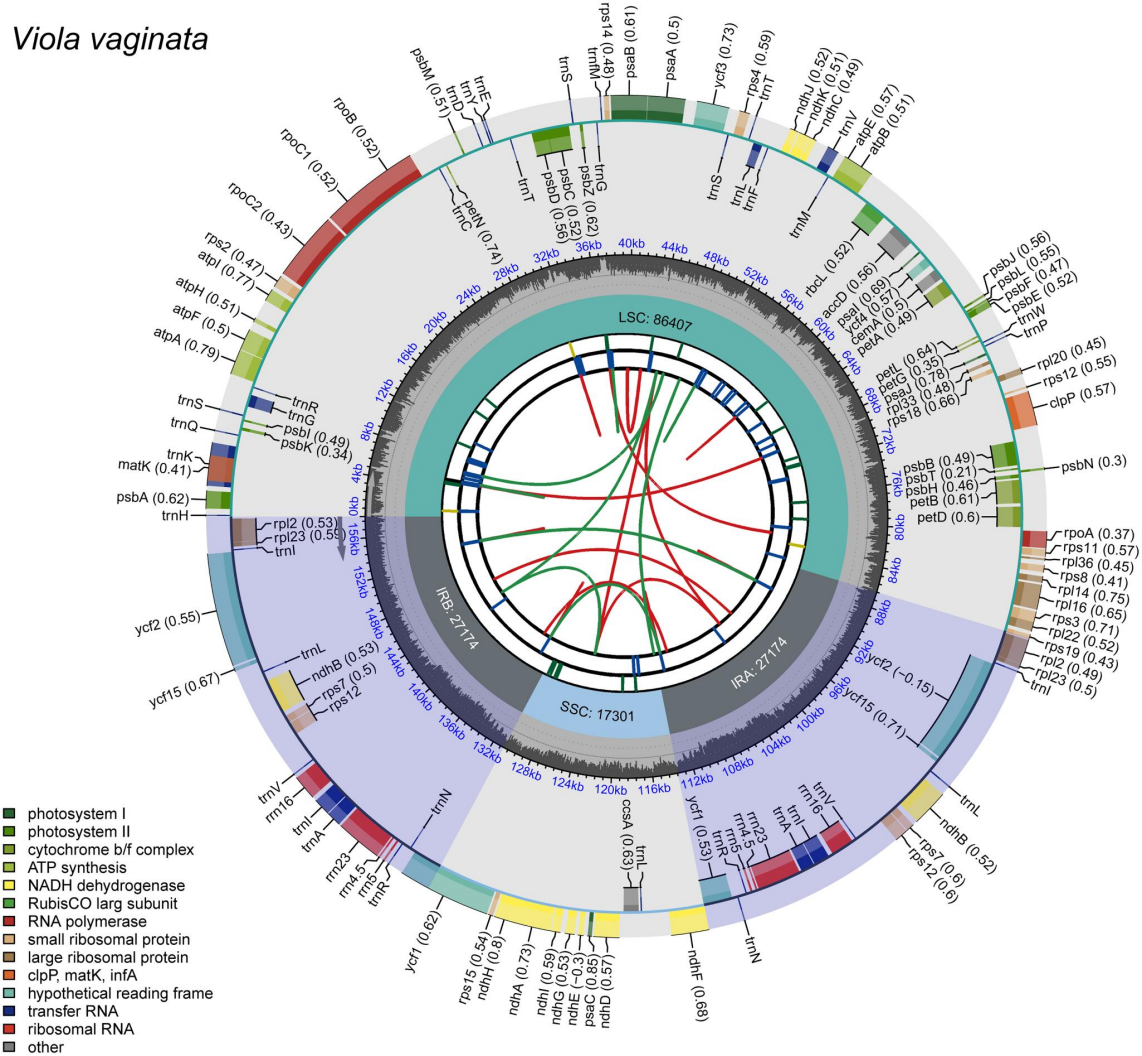
Complete chloroplast genome map of *Viola vaginata*, constructed using CPGView (http://www.1kmpg.cn/cpgview/). the map comprises six tracks. From the center outward: the first track displays dispersed repeats, including direct (red) and palindromic (green) repeats, linked by colored arcs. The second track illustrates long tandem repeats as short blue bars, and the third track depicts short tandem repeats or microsatellite sequences, with each color representing a different type of repeat. The fourth track highlights the small single-copy (SSC) region, inverted repeat regions (IRa and IRb), and large single-copy (LSC) region. The fifth track plots the GC content across the genome. The sixth and outermost track presents the genes, color-coded by functional classification and annotated with optional codon usage bias in parentheses, where applicable. The transcription directions of the genes are indicated by their orientation, with genes on the inner and outer parts of the map being transcribed clockwise and anticlockwise, respectively. A legend detailing the functional classification of genes is shown in the bottom left corner.

We obtained 20,044,410 raw reads, with 96.40% being Q20 or higher. The assembled complete chloroplast genome of *V. vaginata* (LC802718) was 156,056 bp in length, with an overall GC content of 36.3%, and an average coverage of 1524.19 (Figure 2 and Supplementary Figure 1). It exhibited a typical quadripartite structure comprising one large single-copy (LSC) region of 86,407 bp, one small single-copy (SSC) region of 17,301 bp, and two inverted repeats (IR) regions (IRa and IRb, each 27,174 bp) (Figure 2). A nucleotide at position 44,451 in the NOVOPlasty result was corrected to T, as over 75% of mapped reads supported this base. This correction was made to align with the expected haploidy of the chloroplast DNA, which should not exhibit heterozygosity.

The chloroplast genome, excluding duplicate genes, comprised 111 unique genes: 77 protein-coding, 30 transfer RNA, and 4 ribosomal RNA genes. Among these, 8 protein-coding, 7 transfer RNA, and all 4 ribosomal RNA genes were duplicated, with each gene represented in both the IRa and IRb regions. Eleven protein-coding genes were cis-spliced, nine (*atpF*, *rpoC1*, *petB*, *petD*, *rpl16*, *rpl2*, *ndhB*, *ycf1*, and *ndhA*) with one intron, and two (*ycf3* and *clpP*) with two introns each (Supplementary Figure 2 A). The placement of exon regions in the trans-splicing gene *rps12* was also confirmed (Supplementary Figure 2B).

**Figure 3.**
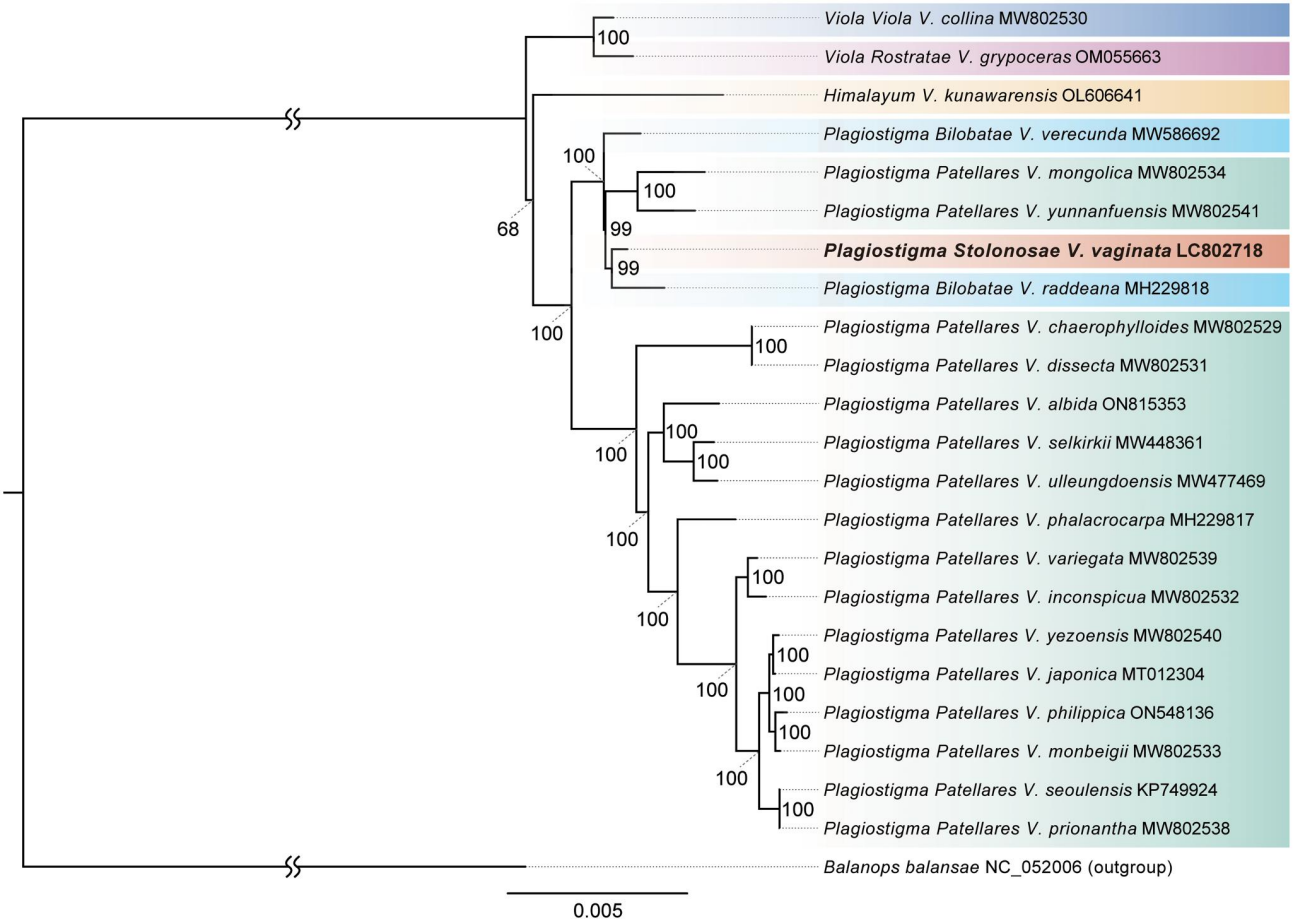
Maximum likelihood phylogenetic tree of 22 *Viola* species and *Balanops balansae* based on chloroplast genome sequences. The following sequences were used: MW802530 (Cao et al. 2022), OM055663 (Park et al. 2023), OL606641 (Zhou et al. 2022), MW586692 (Kwak 2021), MW802534 (Cao et al. 2022), MW802541 (Cao et al. 2022), LC802718 (this study), MH229818 (Cheon et al. 2019), MW802529 (Cao et al. 2022), MW802531 (Cao et al. 2022), ON815353 (Moon and Kim 2023), MW448361 (Go et al. 2022), MW477469 (Go and Yoo 2023), MH229817 (Cheon et al. 2019), MW802539 (Cao et al. 2022), MW802532 (Cao et al. 2022), MW802540 (Cao et al. 2022), MT012304 (Cheon et al. 2020), ON548136 (Cao et al. 2022), MW802533 (Cao et al. 2022), KP749924 (Cheon et al. 2019), MW802538 (Cao et al. 2022), and NC_052006 (Jin et al. 2020). The tips of the tree indicate the section name, subsection name (if applicable), species name, and GenBank accession number. Tip names are based on the classification from Marcussen et al. (2022). Node values represent bootstrap values obtained from 1000 iterations.

The refined alignment length for phylogenetic analysis was 142,005 bp. Most of the excluded regions were intergenic regions and introns, and all gaps were removed (Supplementary dataset 1). The phylogenetic analysis supported the monophyly of the section *Plagiostigma* with 100% bootstrap probability, further dividing into two clades (Figure 3). One clade included 14 species from subsection *Patellares*, and the other consisted of five species: *V. vaginata* from subsection *Stolonosae*, two from subsection *Bilobatae*, and two from subsection *Patellares*.

## Discussion and conclusion

In this study, we sequenced the chloroplast genome of *V. vaginata* from subsection *Stolonosae*, revealing its phylogenetic position for the first time. The genome mirrors the gene number and structures seen in closely related *Viola* species. Aligning with Marcussen et al. (2022), we confirmed the monophyly of the section *Plagiostigma*, though we found differing phylogenetic relationships within the section (Figure 3). Our analyses revealed two clades: a mixed clade of subsections *Stolonosae*, *Bilobatae*, and *Patellares*, and a second solely comprising *Patellares*. Contrastingly, the nuclear ITS phylogenetic tree suggested closer relations between *Patellares*, *Diffusaem*, and *Bilobatae*, with *Stolonosae* as a distinct clade (Marcussen et al. 2022), indicating potential discrepancies due to chloroplast capture from past hybridization events. Cao et al. (2022) reported that *V. mongolica* and *V. yunnanfuensis*, from subsection *Patellares*, form a mixed clade with *Bilobatae* based on chloroplast genomes. Our data also include *Stolonosae* in this clade. Notably, species from subsections *Diffusaem* and *Australasiaticae* have not yet been sequenced for their chloroplast genomes. To elucidate a more comprehensive understanding of the phylogenetic relationships within section *Plagiostigma* and to clarify the taxonomic classification, further extensive research is essential, incorporating both chloroplast and multiple nuclear genes across more species.

## Ethical approval

The subject of this research, *Viola vaginata*, is not listed as an endangered or protected species and does not fall under any restrictions requiring specific collection permits for scientific studies. All research activities were conducted in accordance with applicable local and institutional guidelines.

## Supplementary Material

SupplementaryFigure_1_R1_ver20240421_600dpi6inch.tif

SupplementaryFigure_2_R1_ver20240421_600dpi6inch.tif

## Data Availability

The genome sequence data that support the findings of this study are openly available in the DNA Data Bank of Japan Center (DDBJ Center; http://www.ddbj.nig.ac.jp DDBJ) under the accession numbers LC802718. The associated BioProject, SRA, and Bio-Sample numbers are PRJDB17608, DRR532031, and SAMD00749683, respectively.
