# Supplementary figures and images for: The complete chloroplast genome of Viola vaginata (Violaceae), an endemic species of the snowy region in Japan
Source: Mitochondrial DNA B Resour. 2024 Dec 24;10(1):47–51. doi: 10.1080/23802359.2024.2444595 (PMC11703538; doi:10.1080/23802359.2024.2444595)

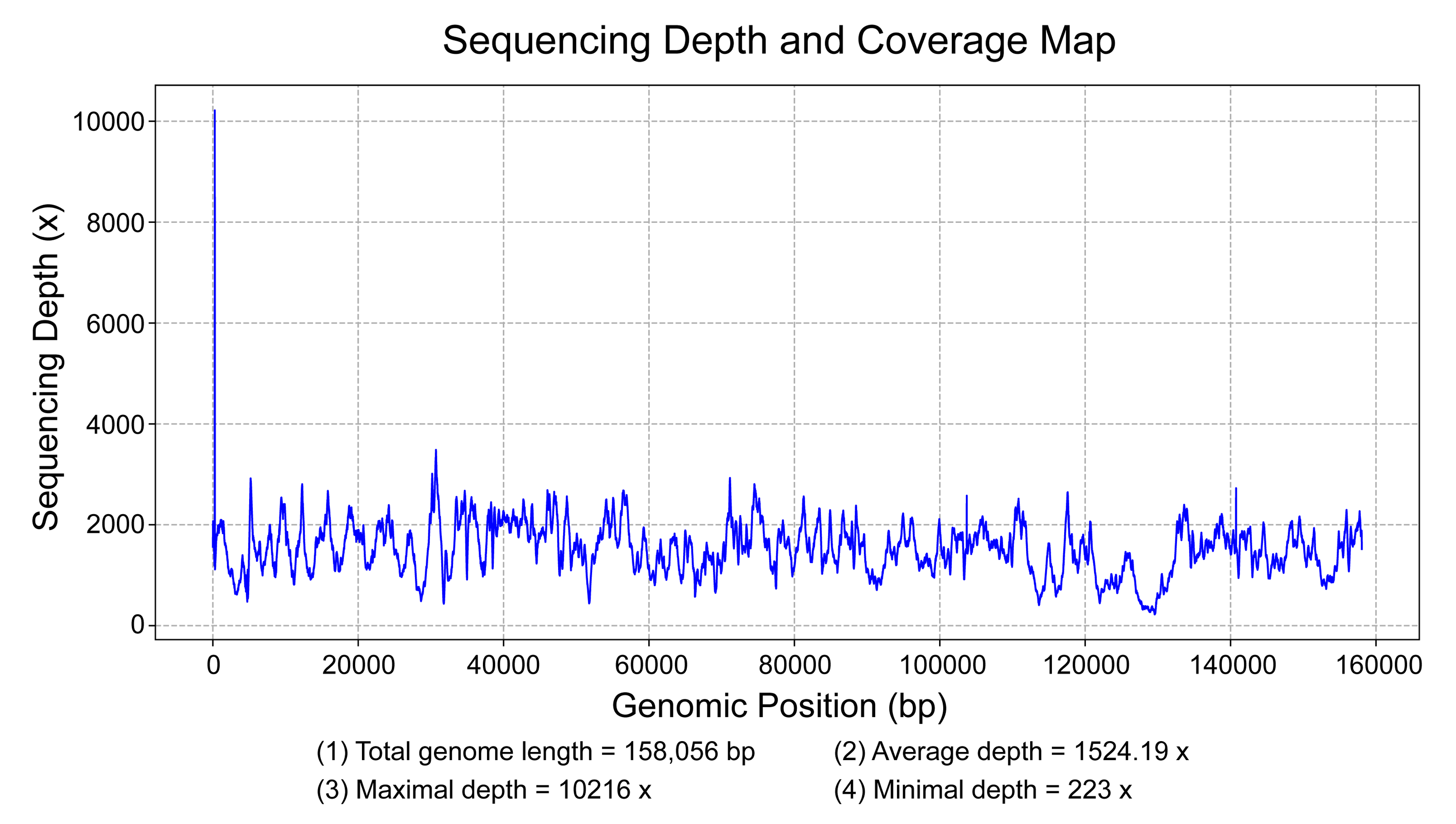

Supplement: SupplementaryFigure_1_R1_ver20240421_600dpi6inch.tif [file TMDN_A_2444595_SM2822.tif]

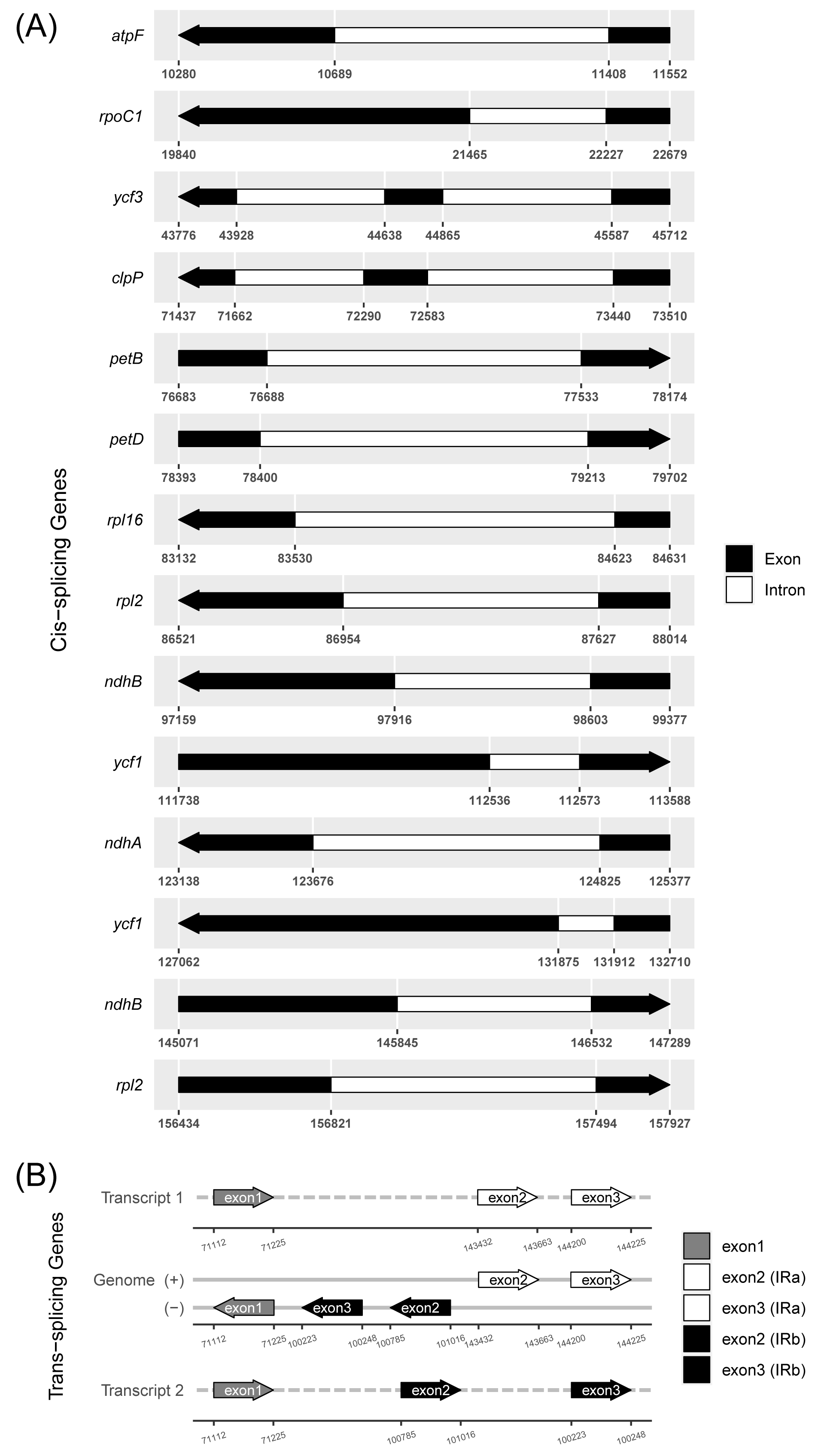

Supplement: SupplementaryFigure_2_R1_ver20240421_600dpi6inch.tif [file TMDN_A_2444595_SM2821.tif]
